# Basal ganglia alterations in amyotrophic lateral sclerosis

**DOI:** 10.3389/fnins.2023.1133758

**Published:** 2023-04-05

**Authors:** Veronica Castelnovo, Elisa Canu, Filippo De Mattei, Massimo Filippi, Federica Agosta

**Affiliations:** ^1^Neuroimaging Research Unit, Division of Neuroscience, IRCCS San Raffaele Scientific Institute, Milan, Italy; ^2^ALS Center, SC Neurologia 1U, AOU Città della Salute e della Scienza of Torino, Turin, Italy; ^3^Neurology Unit, IRCCS San Raffaele Scientific Institute, Milan, Italy; ^4^Neurorehabilitation Unit, IRCCS San Raffaele Scientific Institute, Milan, Italy; ^5^Neurophysiology Service, IRCCS San Raffaele Scientific Institute, Milan, Italy; ^6^Vita-Salute San Raffaele University, Milan, Italy

**Keywords:** amyotrophic lateral sclerosis, basal ganglia, biomarkers, caudate, putamen, globus pallidus, MRI, connectivity

## Abstract

Amyotrophic lateral sclerosis (ALS) has traditionally been associated with brain damage involving the primary motor cortices and corticospinal tracts. In the recent decades, most of the research studies in ALS have focused on extra-motor and subcortical brain regions. The aim of these studies was to detect additional biomarkers able to support the diagnosis and to predict disease progression. The involvement of the frontal cortices, mainly in ALS cases who develop cognitive and/or behavioral impairment, is amply recognized in the field. A potential involvement of fronto-temporal and fronto-striatal connectivity changes in the disease evolution has also been reported. On this latter regard, there is still a shortage of studies which investigated basal ganglia (BG) alterations and their role in ALS clinical manifestation and progression. The present review aims to provide an overview on the magnetic resonance imaging studies reporting structural and/or functional BG alterations in patients with ALS, to clarify the role of BG damage in the disease clinical evolution and to propose potential future developments in this field.

## 1. Introduction

Amyotrophic lateral sclerosis (ALS) – the most common motor neuron disease – is a rare and fatal neurodegenerative disorder of the motor system and its wider connections, in which both upper and lower motor neurons are affected (Hardiman et al., 2017). The clinical phenotype is heterogeneous. Beyond motor impairment, cognitive and/or behavioral disturbances are observed in about 50% of patients and a concomitant frontotemporal dementia (FTD) is present in about 13% of cases (Hardiman et al., 2017; Pender et al., 2020).

Amyotrophic lateral sclerosis has traditionally been associated with magnetic resonance imaging (MRI) alterations of motor brain regions, including corticospinal tract (CST) and corpus callosum hyperintensities on T2-weighted images (Fabes et al., 2017), hypointensity of the cortical band along the precentral gyrus (Roeben et al., 2019) cortical thinning of the primary motor cortex on T1-weighted images (Agosta et al., 2012), and CST and body of the corpus callosum microstructural changes using diffusion tensor imaging (Agosta et al., 2010; Muller et al., 2016). Several MRI studies demonstrated also cerebral extra-motor changes in ALS patients, such as structural and functional alterations of frontal and temporal regions (Agosta et al., 2018). Frontotemporal abnormalities in ALS have been associated especially, but not exclusively, with cognitive and/or behavioral impairment and the more advanced disease stage (Lillo et al., 2012; Trojsi et al., 2013; Agosta et al., 2016; Castelnovo et al., 2022).

The term basal ganglia (BG) refers to archaic brain structures including the striatum, which is the main input region of the BG and it is divided in caudate, putamen and accumbens, the globus pallidus, the substantia nigra, and the subthalamic nucleus, with thalamus being one of the major relay stations to the cortex (Burbaud et al., 2022). BG function was originally considered to be associated with motor control; however, it is now well known that these structures have a crucial role also in cognition and behavior (Obeso et al., 2014). According to the ALS neuropathological staging system based on the distribution patterns of phosphorylated 43-kDa TAR DNA-binding protein (pTDP-43), the large neurons of the thalamic nuclei that project to layer IV of the cerebral cortex develop pTDP-43 aggregates in stage 2, while pTDP-43 pathology extends to caudate, putamen, and especially the ventral striatum (i.e., accumbens) in stage 3 of the disease (Brettschneider et al., 2013). MRI studies in ALS patients show structural BG alterations (Bocchetta et al., 2021) as well as an altered connectivity between BG and frontal regions and networks (Masuda et al., 2016; Basaia et al., 2020; Castelnovo et al., 2020, 2022; Cividini et al., 2021). However, only a few studies which reported evidence of BG alterations in ALS investigated their potential role in clinical manifestations and progression.

The present review aims to provide an overview on the MRI studies which reported BG alterations in patients with ALS, to clarify the role of BG damage in the disease clinical evolution and to propose potential future developments in this field.

## 2. Materials and methods

### 2.1. Inclusion and exclusion criteria

Articles were selected according to predefined inclusion criteria: (a) studies on ALS patients; (b) studies on BG, including thalamus; (c) studies using structural and functional MRI; and (d) studies available in English and in full-text. We excluded: (a) studies on animals and other clinical populations; (b) case reports, reviews, or meta-analyses.

### 2.2. Search strategy

**FIGURE 1:**
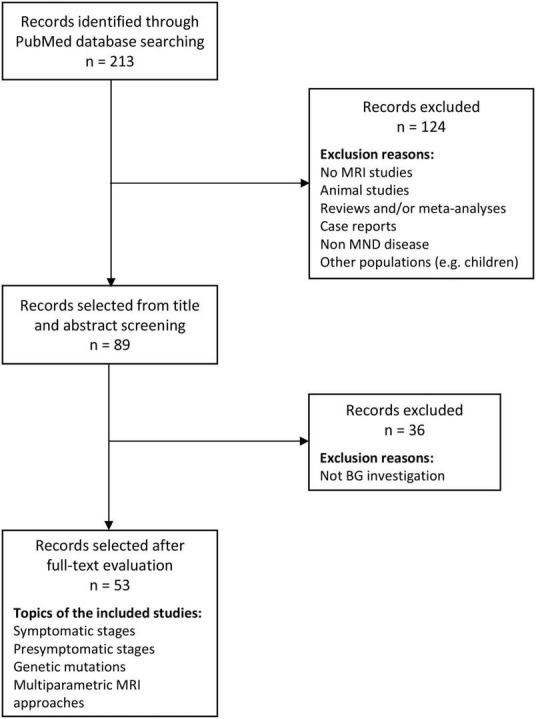
Flow chart of the reviewing process. MND, motor neuron disorders; MRI, magnetic resonance imaging.

**FIGURE 2:**
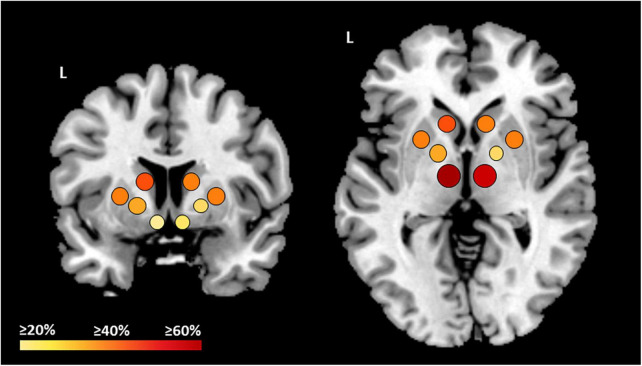
Overview on the 53 reported studies which observed alterations of BG in ALS patients. The color shades and size of the dots represent the amount of studies which observed structural and/or functional alterations in each structure. Bigger and red dots mean ≥60% studies, orange and medium dots mean ≥40% studies, and small and yellow dots mean ≥20% studies. L, left.

A formal literature review was performed using PubMed database on relevant articles, published in peer-reviewed journals until October 2022. The final search line was the following: (“basal ganglia”) OR (“putamen”) OR (“caudate nucleus”) OR (“globus pallidus”) OR (“striatum”) OR (“accumbens”) OR (“thalamus”) OR (“subthalamic nucleus”) AND (“amyotrophic lateral sclerosis”) OR (“ALS”) OR (“motor neuron disease”) OR (“MND”) AND (“MRI”) OR (“magnetic resonance imaging”) OR (“VBM”) OR (“fMRI”) OR (“cortical thickness”) OR (“DTI”) OR (“diffusion tensor imaging”) OR (“seed-based”) OR (“resting-state”) OR (“resting state”) OR (“white matter”) OR (“gray matter”) OR (“TBSS”) OR (“Tract-Based Spatial Statistics”) OR (“Tractography”) OR (“Connectivity”) OR (“Connectome”) OR (“graph-based”). We obtained 213 articles. After abstract and full-text screening, 53 articles were considered eligible for the aim of the study and were included in the present review (Figure 1). All these studies and their main features are reported and discussed below. Furthermore, a summary of the 53 selected studies together with the main representative cognitive/behavioral instruments used to capture BG alterations are reported in Supplementary Table 1. Highlights of the findings and the glossary of the reported imaging techniques and measures are shown in Tables 1, 2, respectively. Figure 2 provides an overview on the number of studies which observed alterations in each BG structure.

**TABLE 1:** Highlights of the findings.

Gray matter damage	• The majority of studies demonstrated an involvement of BG in ALS, particularly in patients with cognitive/behavioral impairment. • Measures of iron deposition, metabolism and atrophy of BG are able to distinguish ALS cases from controls with relatively high accuracy. • Longitudinal studies considering 6–9 months of follow-up evidenced a progressive reduction of the striatum volume, mainly in ALS patients with a fast progression. • Longitudinal studies considering 24 months of follow-up evidenced a widespread progressive reduction of BG volume, including caudate, pallidum, putamen, and thalamus in ALS patients. • ALS patients with C9orf72 expansion show more extensive involvement of BG structures than those who do not carry a C9orf72 mutation.
Microstructural changes	• DTI studies revealed lower microstructural integrity of the striatum in ALS when compared to controls, mainly in cases with cognitive/behavioral involvement and/or fast progression. • The structural connectivity is reduced between the head of caudate, the dorsomedial frontal and the lateral orbitofrontal cortices in ALSci and ALS-FTD patients. • Longitudinal studies considering 6–24 months of follow-up evidenced progressive microstructural changes in the bilateral thalamus of ALS patients.
Functional MRI studies	• Compared with ALScn, ALSci, and ALS-FTD patients show hypoconnectivity in the ventral attentional network. • At the 0.03–0.06 frequency bands ALS patients exhibit increased BG connectivity. • Altered RS-FC between BG and orbitofrontal and temporal regions is associated with worse cognitive performances in ALS. • Altered RS-FC between striatum and SMA and cerebellum is related with greater disease severity and shorter disease duration. • Most fMRI studies reported an increased activation of caudate and putamen when ALS patients performed a motor task. • The altered functional connectivity between GP and frontal and temporal cortices is related with worse emotional processing in ALScn.
Graph analysis and connectome	• So far, few studies investigated structural and functional connectivity in ALS using graph analysis and connectomics. • Compared to controls, ALS showed reduced mean structural local efficiency and longer path length in BG, as well as reduced microstructural integrity within the BG structural network. • Distruction of the structural connectome, which includes BG, occurs also in ALScn and precedes functional alterations. • Nodal efficiency of the left caudate is decreased in ALSci.

ALS, amyotrophic lateral sclerosis; ALSci, ALS with cognitive impairment; ALScn, ALS cognitively normal; ALS-FTD, ALS-frontotemporal dementia; BG, basal ganglia; DTI, diffusion tensor imaging; fMRI, functional magnetic resonance imaging; GP, globus pallidus; RS-FC, resting state-functional connectivity; SMA, supplementary motor area.

**TABLE 2:** Glossary of the technical terms used in this review.

Gray matter	
Voxel based morphometry	Neuroimaging technique that investigates voxel-wise gray or white matter differences between groups.
Volumetric analysis	An approach that processes T1-weighted images for obtaining brain structure volumes.
Cortical thickness	Brain morphometric measure derived from T1-weighted images that describes the thickness of gray matter layers in the cerebral cortex.
Tensor-based morphometry	Neuroimaging technique used to localize regions of structural differences between groups or changes over time in subjects’ repeated scans, based on deformation fields that align one image (e.g., a template) to another (e.g., individual map).
**Microstructure**	
Tractography	Three-dimensional modeling technique used to represent neural tracts using diffusivity data collected by diffusion tensor imaging (DTI) to evaluate the presence of continuous axons that traverse multiple voxels.
Tract-based spatial statistics	Automated and observer-independent approach for a voxel-wise analysis of white matter microstructural integrity.
Fractional anisotropy	A DTI-derived measure of the diffusion of water molecules which reflects fiber density, axonal diameter, and myelination in white matter. It ranges from 0, isotropic movement of water molecules (unrestricted in all directions), to 1, anisotropic movement of water molecules (diffusion occurs only along one axis and is fully restricted along all other directions).
Mean diffusivity	DTI-derived measure of the average directionally independent amplitude of water diffusion within brain tissue.
Axial diffusivity	DTI-derived measure of water molecule diffusion parallel to the fibers within the voxel of interest.
Radial diffusivity	DTI-derived measure of water molecule diffusion perpendicular to the fibers within the voxel of interest.
ROI analysis	Approach that involves the extraction of signal from specified regions of interest.
**Functional MRI**	
Resting state fMRI	fMRI technique based on the analysis of low-frequency fluctuations of the blood oxygenation level dependent (BOLD) signal in the absence of tasks or external stimuli (“at rest”). It is used to evaluate regional brain interactions that occur in a resting state.
Task-based fMRI	fMRI technique based on the analysis of low-frequency fluctuations of the blood oxygenation level dependent (BOLD) signal during the performance of a specific action by the patient. It is used to map regional brain activity that occurs in response of cognitive, perceptual or motor manipulations.
Dynamic causal modeling (applied to fMRI)	Bayesian scheme used to construct and compare generative models of measured blood oxygen level-dependent signals to compute neuronal effective connectivity between brain regions.
Fractional amplitude of low-frequency fluctuations	The ratio between the average value of frequency fluctuations within a specific frequency band (from 0.01 to 0.08 Hz) and the average of the whole range of frequencies. In this way, it specifically reports the spontaneous resting state activity of each region with respect to the rest of activity measured in the brain.
Effective connectivity	Effective connectivity is defined as the influence that a node exerts over another node under a network model of causal dynamics and is inferred from a model of neuronal integration, which defines the mechanisms of neuronal coupling.
Voxel-mirrored homotopic connectivity	fMRI technique to analyze the functional homotopy between the two hemispheres, by computing the connectivity between each voxel in one hemisphere and its mirrored counterpart in the other.
**Graph analysis and connectome**	
Graph theory-based analysis	Graph theory-based approaches describe brain as a complex network represented graphically as a set of nodes and edges, where nodes indicate anatomical elements (e.g., brain regions), and edges represent the relationships between nodes (e.g., connectivity).
Connectome	Connectomics is the study of the connections in the central nervous system. The entire set of connections that constitute the brain is known as connectome.
Local efficiency	Measure that expresses the level of local connectedness of a network, with high levels of clustering interpreted as high levels of local organization of the network.
Path length	Distance between two nodes, measured as the number of edges that must be crossed to go from one node to another. It is inversely related to the global efficiency.
Clustering coefficient	The clustering coefficient describes the tendency of nodes to form local triangles, providing insights into the local organization of the network.

## 3. Results

### 3.1. Structural MRI studies

#### 3.1.1. Gray matter damage

Several studies assessing gray matter (GM) loss in patients with sporadic ALS reported alterations of caudate head (Agosta et al., 2009; Bede et al., 2013, 2016, 2018; Menke et al., 2014; Machts et al., 2015; Masuda et al., 2016; Senda et al., 2017; Alruwaili et al., 2018; Christidi et al., 2018b; Tae et al., 2020; Ahmed et al., 2021; Canna et al., 2021), both in patients with slow and fast disease progression (Agosta et al., 2009; Senda et al., 2017), putamen (Agosta et al., 2009; Machts et al., 2015; Buhour et al., 2017; Kim et al., 2017; Bede et al., 2018; Christidi et al., 2018a,b; Tae et al., 2020; Ahmed et al., 2021; Canna et al., 2021), globus pallidus (Machts et al., 2015; Bede et al., 2018; Tae et al., 2020; Ahmed et al., 2021; Canna et al., 2021; Castelnovo et al., 2021; Leoni et al., 2022), accumbens (Bede et al., 2013, 2016, 2018; Machts et al., 2015; Finegan et al., 2019), and thalamus (Chang et al., 2005; Thivard et al., 2007; Bede et al., 2013, 2016; Machts et al., 2015; Masuda et al., 2016; Senda et al., 2017; Branco et al., 2018; Christidi et al., 2018a; Menke et al., 2018; Tu et al., 2018; Finegan et al., 2019; Chipika et al., 2020; Ahmed et al., 2021; Liu et al., 2021; Li et al., 2022). A recent cross-sectional study, which stratified ALS patients in different disease stages based on the King’s clinical staging system, found greater atrophy in the bilateral thalamus of patients at King’s stage 3, compared to controls and ALS patients at King’s stage 1 or 2 (Liu et al., 2021). Reduced local shape distances of the putamen (reflecting the presence of atrophy) were associated with a higher rate of disease progression (Tae et al., 2020), while reduced accumbens volumes correlated with greater apathy (Machts et al., 2015). The extent of BG involvement in ALS was also related to the cognitive/behavioral status of patients, which only a restricted number of studies took into account (Chang et al., 2005; Bede et al., 2013, 2018; Machts et al., 2015; Branco et al., 2018; Christidi et al., 2018b; Tae et al., 2020; Ahmed et al., 2021).

More in detail, a cross-sectional study which compared GM volumes of ALS patients with normal cognition (ALScn), patients with ALS-FTD and patients with behavioral variant FTD (bvFTD) demonstrated atrophy of the bilateral globus pallidus, putamen and thalamus in all groups of patients compared to controls, with a lesser degree of involvement in ALScn cases (Ahmed et al., 2021). In this study, a reduced volume of the globus pallidus, caudate, putamen, and thalamus was associated with lower global cognition scores, while thalamic atrophy was associated with poorer executive function assessed using trail making test (Ahmed et al., 2021). Concerning behavior, a reduced volume of nucleus accumbens, putamen and thalamus was associated with an increased number of behavioral disturbances as odd beliefs, stereotypic and ritualistic behaviors and reduced motivation, while a reduced volume of globus pallidus only was related to an increased number of odd beliefs (Ahmed et al., 2021). Another study in ALS patients, of whom 10% were ALS-FTD and 6 ALS patients with cognitive impairment (ALSci), found volume reduction and differences in the shape of the volumes of the left caudate, bilateral accumbens and thalamus, and total BG of all ALS compared to controls (Bede et al., 2013).

In contrast to the previously described studies, others revealed atrophy of putamen, globus pallidus, caudate, accumbens, and thalamus exclusively in ALS-FTD patients and no BG volume reduction in ALScn patients (Machts et al., 2015; Masuda et al., 2016; Bede et al., 2018; Tae et al., 2020). Alterations in the caudate and putamen were confirmed by a voxel-based morphometry (VBM) analysis in ALS patients with cognitive/behavioral deficits (ALSci/bi) and ALScn patients, in which reduced GM volume of the left caudate and putamen was observed only in ALSci/bi (Christidi et al., 2018b). Similarly, another VBM study which compared ALSci and ALScn patients reported reduced GM volume of the right putamen in the former group (Kim et al., 2017). Furthermore, a volumetric analysis demonstrated that ALSci patients had smaller volumes of the left thalamus compared to ALScn and that reduced thalamic volumes were associated with higher scores at the Amyotrophic Lateral Sclerosis Cognitive Behavioral Screen (Branco et al., 2018). A recent study in ALScn patients reported no brain volumetric differences between patients and controls; however, in ALScn patients, significant correlations were observed between lower performances in disgust recognition and the Edinburgh Cognitive and Behavioral ALS Screen (ECAS) and reduced volume of the left globus pallidus (Castelnovo et al., 2021).

Interestingly, a ROC analysis combined three quantitative imaging markers, namely iron deposition, basal neuronal metabolism, and regional atrophy, to assess the ability in discriminating ALS patients from controls and from patients with primary lateral sclerosis (PLS) (Canna et al., 2021). The analysis demonstrated that the involvement of bilateral motor cortex, bilateral caudate, right hippocampus, right putamen, right globus pallidus, and right amygdala differentiated ALS patients and controls with a mean accuracy of 0.68, and specifically right and left caudate, right putamen and right globus pallidus showed a range of accuracy between 0.66 and 0.69 (Canna et al., 2021). Another study showed reduced GM volume and increased iron deposition in the bilateral thalamus in ALS patients compared to controls, and a positive relationship between increased iron deposition and disease severity (Li et al., 2022).

Relatively few longitudinal MRI studies assessed BG structural alterations in sporadic ALS. In a large cross-sectional and longitudinal study in ALS patients (not cognitively characterized), regional volume change over time was found in both thalami, and an association was observed between smaller BG volume at baseline and shorter survival (Westeneng et al., 2015). Another longitudinal study reported decreased GM volume over a 6-month follow-up in the head of caudate and thalamus bilaterally in ALS patients (Menke et al., 2014). More recently, the same group performed another longitudinal study with a longer observation period and reported decreased GM volume in ALS over a 24-month follow-up also in globus pallidus and putamen (Menke et al., 2018). These findings are in line with a longitudinal tensor based morphometry study showing, over 9 months, a greater involvement of right caudate and putamen of ALS patients with rapid progression compared to those with non-rapid progression (Agosta et al., 2009).

Concerning genetic cases, several studies reported BG alterations in ALS patients carrying C9orf72 mutation (C9ALS) (Bede et al., 2013; Westeneng et al., 2016; De Vocht et al., 2020; Ahmed et al., 2021; Spinelli et al., 2021). Changes in the caudate and thalamus are consistent findings in most of them (Bede et al., 2013; Walhout et al., 2015; Westeneng et al., 2016; Spinelli et al., 2021). More in detail, compared to healthy controls, significant volume reductions were observed in the bilateral thalamus, left caudate and putamen in a sample of C9ALS patients of whom 67% had ALS-FTD, 22 ALSci, and 11 ALScn (Bede et al., 2013), and in the bilateral thalamus, putamen and accumbens, right caudate and left pallidum in a sample of C9ALS patients of whom 21% had ALS-FTD (Walhout et al., 2015). GM atrophy was limited to the right accumbens and bilateral thalamus in a C9ALS-FTD cohort compared to controls (Bede et al., 2018), while when compared with a sample of sporadic ALS–FTD patients, C9ALS-FTD cases presented reduced GM volumes of the bilateral putamen, accumbens, pallidum, and thalamus (Ahmed et al., 2021). Compared with non-carriers ALS, C9ALS patients (not classified in terms of cognitive status) showed significant smaller volume reduction in the bilateral thalamus, caudate, putamen, and accumbens (Westeneng et al., 2016). Interestingly, reduced BG volume, mainly of the bilateral thalamus, left caudate, and putamen, has been observed also in presymptomatic C9orf72 carriers compared to non-carriers (Walhout et al., 2015; De Vocht et al., 2020). Instead, a volumetric analysis performed in presymptomatic and symptomatic carriers of the Pro56Ser variant of the gene-encoding vesicle-associated membrane-protein-associated protein B (VAPB), revealed significant atrophy of the globus pallidus exclusively in the symptomatic carriers when compared to controls (Leoni et al., 2022).

#### 3.1.2. Microstructural changes

Microstructural changes have been extensively investigated in patients with sporadic ALS using diffusion tensor imaging (DTI). Several DTI studies which used tract-based spatial statistics (TBSS) consistently showed lower fractional anisotropy (FA) and/or higher mean diffusivity (MD) of the globus pallidus (Langkammer et al., 2010; Bede et al., 2013; Sharma et al., 2013), putamen (Langkammer et al., 2010; Sharma et al., 2013), accumbens (Bede et al., 2013), caudate (Langkammer et al., 2010; Sharma et al., 2013; Barbagallo et al., 2014; Masuda et al., 2016; Senda et al., 2017), thalamus (Sach et al., 2004; Thivard et al., 2007; Sharma et al., 2013; Barbagallo et al., 2014; Senda et al., 2017), and thalamic radiations (Masuda et al., 2016; Alruwaili et al., 2018; Tu et al., 2018) in ALS patients compared to controls, with a more severe and widespread involvement of the caudate and thalamus in rapid progressors compared to slow progressor patients (Senda et al., 2017). Greater microstructural integrity of the globus pallidus and caudate in patients was associated with higher maximum rate of foot- and finger taps, and higher FA values of the putamen were related to higher bulbar muscle-movement rates of syllable repeats (Sharma et al., 2013). Furthermore, higher MD values (reflecting low microstructural integrity) of the putamen were related with greater disease severity, while higher MD values of the caudate correlated with lower global cognition scores (Sharma et al., 2013). Higher MD of the caudate was also found to be related with lower scores in tests assessing frontal functions, while higher MD of both caudate and thalamus was related with a longer disease duration (Barbagallo et al., 2014). A study in which half of the ALS sample developed signs of upper motor neuron involvement after the time of MRI investigation, observed reduced FA exclusively in the thalamus of patients compared to controls (Sach et al., 2004).

A longitudinal study reported decreased FA of the right thalamus and increased MD, axial diffusivity (AD), and radial diffusivity (RD) of bilateral thalamus and caudate in ALS patients over a 24-month follow-up (Menke et al., 2018). Another longitudinal study focused on thalamus and parcellated it into distinct regions based on structural thalamo-cortical connectivity (Tu et al., 2018). At the first MRI, compared to controls, ALS patients showed increased MD, AD, and RD in thalamic parcellations connected with the left frontal lobe, bilateral premotor cortex, bilateral motor cortex, right somatosensory cortex, and bilateral parietal lobe; after 6 months they showed increased MD, RD, and AD exclusively in the right frontal lobe and bilateral temporal lobe thalamic areas (Tu et al., 2018). Increased RD in thalamic parcellations connected with the right premotor and motor cortex and right somatosensory cortex at the first MRI visit was related to increased disease severity and longer disease duration (Tu et al., 2018).

Diffusion tensor imaging alterations of BG in ALS were related to variable degree of cognitive/behavioral impairment. A TBSS analysis revealed a widespread decreased FA in the superior thalamic radiation, regions surrounding the head of the caudate and the stria terminalis in ALSci and ALS-FTD, with a trend toward damage in ALScn patients (Masuda et al., 2016). In the same study, a decreased structural connectivity between the caudate head and the dorsomedial frontal and the lateral orbitofrontal cortices were found in all ALS groups (Masuda et al., 2016). A study assessing microstructural correlates of ECAS in an ALS sample of whom 11% had ALSci, 3% had ALScibi, and 17% had ALSbi, found increased AD and MD/RD of the mediodorsal nucleus of the thalamus in relation to lower scores at the ALS non-specific and memory ECAS scores (Trojsi et al., 2019). A study focusing on thalamo-cortical structural connections in ALS demonstrated that compared to controls, ALScn patients presented decreased FA values of the connections between bilateral premotor and motor pathways and bilateral thalamus (Zhang J. Q. et al., 2017). Furthermore, decreased FA values of the tracts connecting thalamus and bilateral motor cortices were related to a longer disease duration (Zhang J. Q. et al., 2017). Decreased FA of the left anterior thalamic radiation was observed in ALS patients with pathological laughing (Christidi et al., 2018a).

Finally, compared to controls, an ALS sample in which 10% had ALS-FTD and 6% had ALSci presented diffusivity alterations in the accumbens and globus pallidus (Bede et al., 2013). In the same study, C9ALS patients showed reduced thalamic FA compared to controls and increased thalamic AD and MD compared to C9orf72-negative patients (Bede et al., 2013). A study on asymptomatic C9orf72 carriers observed no DTI significant differences between C9orf72 carriers and non-carriers (Walhout et al., 2015).

### 3.2. Functional MRI studies

#### 3.2.1. Resting state functional MRI

Functional MRI, in particular resting-state functional connectivity (RS-FC) analysis, has provided important insights into the brain functional reorganization in ALS. The presence of altered functional connectivity and activation of BG in ALS patients has been demonstrated by several studies (Konrad et al., 2006; Tessitore et al., 2006; Mohammadi et al., 2015; Ma et al., 2016; Abidi et al., 2020, 2022; Temp et al., 2021; Bharti et al., 2022; Castelnovo et al., 2022).

A recent resting state functional MRI (RS-fMRI) study observed a widespread decreased RS-FC in the default mode network, motor network and ventral attention network in ALScn, ALSci, and ALS-FTD patients compared to controls (Temp et al., 2021). In the ventral attention network, ALSci and ALS-FTD patients showed hypoconnectivity compared with ALScn. Furthermore, in all patients increased RS-FC of the left putamen and thalamus within the ventral attention network was associated with worse shifting ability assessed using the trail making test (Temp et al., 2021). Two studies demonstrated that the abnormal spontaneous neural activity in ALS is frequency specific (Fekete et al., 2013; Ma et al., 2016). The first is a study in which time series were filtered at 0.03–0.06 frequency bands found that in these bands, compared to controls, ALS patients exhibited significant clusters of decreased functional connectivity within the motor and somatosensory cortices and clusters of increased connectivity in the BG and cerebellum (Fekete et al., 2013). However, no significant correlations with clinical measures were observed (Fekete et al., 2013). The second, a cross sectional study which assessed the fractional amplitude of low-frequency fluctuations (fALFF) at different low-frequency bands using RS-fMRI, reported increased fALFF in the right caudate in ALS patients compared to controls, at slow frequency bands 4 and 5, demonstrating that the abnormal spontaneous neural activity in ALS is frequency specific (Ma et al., 2016). However, no significant correlations were observed between BG fALFF and clinical measures (Ma et al., 2016).

A multicentric study which investigated intra-network and inter-network RS-FC in a large sample of ALS patients (not classified in terms of cognitive status) reported higher intra-network RS-FC in several RS circuits, including BG network, in ALS patients compared to controls (Bharti et al., 2022). These findings were associated with a worse performance at ECAS, worse finger and foot tapping scores, and greater disease severity (Bharti et al., 2022). In addition, ALS patients compared to controls showed a higher inter-network RS-FC between the BG and orbitofrontal networks, which were related to lower ECAS ALS-specific score (Bharti et al., 2022). A seed-based FC analysis which investigated the RS-FC of the globus pallidus in ALScn patients showed an increased RS-FC between bilateral globus pallidus and postcentral, supramarginal, and superior temporal gyri, and reduced RS-FC between the left globus pallidus and left middle and inferior temporal gyri and bilateral caudate, with these latter alterations related to worse disgust recognition in patients and controls (Castelnovo et al., 2022). A study which assessed voxel-mirrored homotopic connectivity (VMHC) found reduced VMHC in the putamen in patients with ALS compared to controls (Zhang J. et al., 2017).

A longitudinal study reported FC decreases in ALS patients over a 24-month follow-up between a network comprising both thalami and an area in the visual cortex in relation to an increased severity of the disease (Menke et al., 2018).

#### 3.2.2. Task-based fMRI

A consistent finding across task-fMRI studies is the increased activation of BG during motor tasks in ALS patients compared to controls (Konrad et al., 2006; Tessitore et al., 2006). During a simple visually paced motor task, a greater activation of the right caudate and left putamen was observed in ALS patients with a more severe upper motor neuron involvement compared to those with greater clinical lower motor neuron involvement (Tessitore et al., 2006). In a second study, subjects were required to squeeze the plastic handle of a grip force transducer cued by an acoustic trigger and an increased fMRI activation was reported in the putamen of ALS patients compared to controls (Konrad et al., 2006). The finding of an increased activity of the putamen in patients has been corroborated by a study in which patients, compared to controls, displayed a higher activity in putamen and globus pallidus during a stop-signal task in which subjects had to answer with a button press to a right- or left-pointing black arrow (go-stimuli) (Mohammadi et al., 2015). Another fMRI study observed no activation of the thalamus in ALS patients with bulbar signs, compared to ALS patients without bulbar signs and controls, during a task which required to vertically move the tongue (Mohammadi et al., 2009).

An fMRI experiment in which supplementary motor area (SMA), cerebellum and striatum were defined as *a priori* seed regions showed that, among BG, the caudate presented increased effective connectivity with the cerebellum and decreased effective connectivity with the SMA when ALS patients with predominant upper motor neuron (UMN) involvement performed self-initiated movements (Abidi et al., 2020). Decreased effective connectivity between SMA and striatum was associated with greater disease severity in patients with ALS and more severe LMN involvement, while decreased striato-cerebellar effective connectivity was related with greater disease severity and shorter disease duration in patients with greater UMN involvement (Abidi et al., 2020). The same group recently performed a similar study using dynamic causal modeling and including also posterior parietal cortex (PPC) as seed-region (Abidi et al., 2022). During gait imagination, ALS patients with greater UMN involvement showed decreased effective connectivity from BG to SMA and from SMA to PPC, while patients with greater LMN involvement presented bilateral connectivity between SMA and BG (Abidi et al., 2022).

### 3.3. Graph analysis and connectome

In the last decade, neuroimaging research began to focus on the study of structural and functional connectivity alterations at a whole-brain-system level, rather than on changes in single regions, applying graph theory analysis and connectomics. To date, still few works used these approaches for the study of brain MRI in ALS and some of them reported BG networks alterations (Xu et al., 2017; Basaia et al., 2020; Cividini et al., 2021). A cross-sectional study applied a graph theoretical approach to investigate the functional abnormalities of the cortico-BG network in ALSci patients, and observed decreased nodal efficiency in the left caudate and right thalamus, compared with controls (Xu et al., 2017). A second study used graph analysis and connectomics to assess the global and lobar topologic network properties and regional structural and functional brain connectivity in a large sample of patients with ALS, PLS, progressive muscular atrophy, and controls. Compared to controls, ALS and PLS patients presented reduced mean structural local efficiency, longer path length, and decreased FA in the BG network (Basaia et al., 2020). In ALS patients, a longer path length within BG network was related to disease progression rate, BG FC changes and connections between BG and premotor areas were associated with disease progression, and a greater functional clustering coefficient within the BG network correlated with worse executive functions (Basaia et al., 2020). More recently, the same group performed a similar analysis by stratifying ALS patients according to cognitive and behavioral status (Cividini et al., 2021). The analysis showed that ALScn presented a more focal structural damage within the sensorimotor-BG areas, ALSci/bi patients demonstrated the same structural damage of ALScn patients, together with enhanced FC within sensorimotor areas and decreased FC within frontotemporal and parietal networks. Finally, ALS-FTD patients showed both structural and functional disruption of the frontotemporal and parietal networks, and the typical ALScn structural damage within the sensorimotor-BG areas, suggesting that BG involvement, even if in a different extent, is present in the two extremes of the ALS-FTD spectrum (Cividini et al., 2021).

## 4. Discussion

The present review provides an overview on MRI studies which identified BG alterations in patients with ALS. This work strongly supports that, as many other clinical variants within the frontotemporal lobar degeneration (FTLD) spectrum (Bocchetta et al., 2021), also ALS is characterized by a considerable BG degeneration. Although apparently surprising given the typical absence of extrapyramidal signs in ALS (Rowland and Shneider, 2001), BG degeneration is consistent with several clinical characteristics of the disease.

As such, given BG involvement in voluntary movement and high-order aspects of motor control (Groenewegen, 2003), structural and functional changes in BG were often associated with greater disease severity (Sharma et al., 2013; Menke et al., 2018; Abidi et al., 2020; Bharti et al., 2022; Li et al., 2022), worsening of clinical symptoms, including greater disability and motor decline (Sharma et al., 2013; Bharti et al., 2022), as well as faster disease progression (Basaia et al., 2020; Tae et al., 2020), and shorter survival (Westeneng et al., 2015; Zhang J. Q. et al., 2017).

Specifically, structural alterations of the caudate nucleus and thalamus and a decrease of their GM volume over time are consistent findings, followed by alterations in the putamen and globus pallidus (see paragraph “Gray matter damage”). Along the same lines, both atrophy and microstructural changes in the caudate and putamen have been consistently associated with a higher rate of disease progression (Agosta et al., 2009; Senda et al., 2017; Tae et al., 2020). Also, reduced microstructural integrity of the striatum and thalamus was associated with more severe motor symptoms and with longer disease duration (Sharma et al., 2013).

Resting-state fMRI revealed that increased connectivity within the BG network was associated with worse finger and foot tapping scores, and with greater disease severity (Bharti et al., 2022), and that decreased FC between a network comprising thalamus and the visual cortex was related to the increased severity of the disease observed over a 24-month period (Menke et al., 2018). Furthermore, task-based fMRI studies consistently showed hyperactivation of BG, in particular of the caudate and putamen, upon successful motor task performance (Konrad et al., 2006; Tessitore et al., 2006; Mohammadi et al., 2015). This finding has been interpreted as a mechanism of functional reorganization and compensation for the ongoing loss of motor neurons. In contrast, no activation of the thalamus during vertical tongue movements was observed in ALS patients with bulbar signs compared to those without bulbar signs and with controls (Mohammadi et al., 2009).

Taken together, all these studies show that BG structural and functional alterations are associated with ALS motor symptoms and decline, suggesting BG involvement as a potential marker of a worse disease prognosis.

Besides their involvement in motor control, BG play a fundamental role in pure cognitive and behavioral domains, such as executive functions (Leisman et al., 2014; Saalmann and Kastner, 2015) and affective processing (Castelnovo et al., 2021, 2022). Morphological studies showed that BG are part of three parallel and segregated circuits that are connected to the frontal lobe, namely the sensorimotor, associative, and limbic circuits (Looi and Walterfang, 2013). Given their different roles in cognition, emotion, and motivation, the dysfunction of these circuits may contribute to the typical neuropsychological profile of ALS patients (Bharti et al., 2022).

Structural changes in BG are often associated with the presence of cognitive and/or behavioral symptoms (Machts et al., 2015; Alruwaili et al., 2018; Branco et al., 2018; Ahmed et al., 2021; Castelnovo et al., 2021). Interestingly, BG structural changes have been observed in ALS patients with C9orf72 repeat expansions, where they are associated with cognitive and or behavioral impairment, as well as in presymptomatic C9orf72 carriers when compared to non-carriers (Bede et al., 2013; Walhout et al., 2015; Westeneng et al., 2016; De Vocht et al., 2020; Spinelli et al., 2021). With respect to RS-fMRI changes, several studies reported functional connectivity alterations both within the BG network as well as between BG and frontal, temporal and parietal regions. Notably, these brain changes are associated with specific cognitive and behavioral features, such as executive dysfunctions, failure in emotion recognition, apathy, and odd beliefs (Abidi et al., 2020; Ahmed et al., 2021; Bharti et al., 2022; Castelnovo et al., 2022). Altogether, the association between BG alterations and cognitive/behavioral manifestations is a relevant feature of ALS. Importantly, this evidence is consistent with similar findings characterizing the FTLD literature (Bocchetta et al., 2021). Some instruments investigating ALS specific domains (e.g., ECAS ALS-specific score), executive dysfunctions (e.g., trail making test), emotion recognition (e.g., the Comprehensive Affect Testing System) and behavior (e.g., Amyotrophic Lateral Sclerosis Cognitive Behavioral Screen) are revealed to be more powerful than others in detecting neuropsychological alterations associated with BG changes. However, neuropsychological tests targeted to cognitive and behavioral changes specifically associated with BG damage have not yet been defined, and could be the aim of future research.

Among ALS phenotypes, ALScn patients seem to present with the mildest BG volume and microstructural alteration profile (Branco et al., 2018; Ahmed et al., 2021). However, TBSS, graph analysis and connectome approaches suggested that structural alterations in BG connections are present also in ALScn patients (Masuda et al., 2016; Cividini et al., 2021). These findings add to the notion of BG alterations being involved throughout the ALS spectrum (from ALScn to ALS-FTD). The presence of BG alterations also in ALScn patients (Masuda et al., 2016; Bede et al., 2018; Basaia et al., 2020; Tae et al., 2020; Castelnovo et al., 2022) can imply two not exclusive hypotheses: (1) subtle cognitive/behavioral deficits, which do not satisfy the current criteria, may be present early during the disease course (Masuda et al., 2016; Tae et al., 2020); and (2) BG alterations may be already present before the overt TDP-43 pathologic burden (i.e., during stage 1) (Brettschneider et al., 2013). It is also conceivable that during early disease stages, other neuropathological processes, such as iron deposition, could herald the accumulation of TDP-43 aggregates in BG (De Reuck et al., 2017). Alternatively, the presence of other neuropathological processes, such as fused in sarcoma (FUS) protein alterations, may occur in BG in initial ALS; this hypothesis is supported by the evidence that initial FTLD cases with a FUS pathology have shown caudate atrophy in comparison with controls (Rohrer et al., 2011).

A major limiting factor emerging from our review is the restricted number of studies assessing BG alterations of ALS *in vivo*. Also, most of these studies compare ALS with controls, and only few works investigate the BG differences among ALS clinical and genetic subgroups. In particular, despite the established importance of neuropsychological features in ALS patients, both in terms of prognosis and adherence to treatment, only few studies address this important facet by stratifying the samples according to cognitive/behavioral status. Similarly, many studies did not investigate the relationship between BG damage and other clinical manifestations.

## 5. Conclusion

In conclusion, this review highlights the importance of BG alterations for a more comprehensive pathophysiological understanding of ALS. At the same time, it supports their MRI-based evidence as reliable prognostic indicator of disease severity and progression from the early and even presymptomatic stages. Future studies should favor a multiparametric clinical/imaging approach coupled with longitudinal assessments to further explore BG alterations in ALS.

## Author contributions

VC and EC conceptualized, designed, and drafted the manuscript. FD critically reviewed the manuscript for important intellectual content. MF and FA conceptualized and designed the manuscript and critically reviewed the manuscript for important intellectual content. All authors contributed to the article and approved the submitted version.
