# Supplementary material for: Basal ganglia alterations in amyotrophic lateral sclerosis
Source: Front Neurosci. 2023 Apr 5;17:1133758. doi: 10.3389/fnins.2023.1133758 (PMC10113480; doi:10.3389/fnins.2023.1133758)
Supplement: Supplementary file 1 [file Table_1.docx]

**Supplementary Table 1.** Summary of the 53 studies selected for the reviewing process.

| **Reference** | **Population** | **Methods** | **Main aims** | **Main BG and thalamus findings** | **Main cognitive measures and behavioural instruments** |
| --- | --- | --- | --- | --- | --- |
| Abidi et al., 2020 | - 17 LMN, predominant ALS patients  - 14 UMN, predominant ALS patients  - 14 HC | MRI sequences:  - T1-weighted  - Task-based fMRI (simultaneous right/left ankle dorsiflexion)  Technique:  - ROI based fMRI analysis (seeds: supplementary motor area, striatum and cerebellum) | To characterize brain functional connectivity during a motor task in a cohort of ALS patients stratified for UMN versus LMN disease burden. | - Compared to HC, UMN predominant patients showed increased effective connectivity between the cerebellum and caudate, and decreased connectivity between the SMA and caudate and between the SMA and cerebellum when performing self-initiated movement.  - In UMN predominant patients, a positive correlation was found between clinical variables (ALSFRS-r and disease duration) and striato-cerebellar connectivity.  - In LMN predominant patients, positive correlations were found between clinical variables (ALSFRS-r and disease progression) and SMA-striatum connectivity. | - |
| Abidi et al., 2022 | - 17 LMN, predominant ALS patients  - 14 UMN, predominant ALS patients  - 14 HC | MRI sequences:  - T1-weighted  - Task-based fMRI (motor and visual imagery tasks)  Technique:  - ROI based fMRI analysis (seeds: supplementary motor area, BG [putamen and caudate], cerebellum, posterior parietal cortex).  - Dynamic causal modeling | To investigate connectivity patterns within the gait control and postural control circuits in ALS. | - During gait imagination, ALS patients with greater UMN involvement showed decreased effective connectivity from BG to SMA and from SMA to PPC, while those with greater LMN involvement presented bilateral increased connectivity between SMA and BG. | - |
| Agosta et al., 2009 | - 16 ALS (8 non-rapidly progressing, 8 rapidly progressing)  - 10 HC  Longitudinal assessment: 9 months | MRI sequences:  - T2- weighted TSE  - T1-weighted  Technique:  - Whole-brain tensor based morphometry  - ROI-based tensor based morphometry analysis (frontal and temporal cortex, caudate and putamen) | To investigate GM contraction in patients with ALS using tensor based morphometry. | - Compared to HC, all ALS patients (rapidly and non-rapidly progressing) showed significant progression of GM atrophy in the right BG (i.e., putamen and caudate nuclei).  - Rapidly progressing ALS patients showed further GM loss in the left caudate and right putamen compared with both HC and non-rapidly progressing ALS patients. | - |
| Ahmed et al., 2021 | - 58 bvFTD (17 C9orf72+)  - 41 ALS–FTD (12 C9+)  - 52 ALScn (3 C9+)  - 58 HC | MRI sequences:  - T1-weighted  Technique:  - Volumetry | To determine phenotypic patterns of cortical and subcortical atrophy at initial presentation, and their relationship with cognitive and behavioural disturbances in ALS and FTD. | - Compared with ALScn, both ALS–FTD and bvFTD showed greater atrophy in the bilateral thalamus.  - Compared with bvFTD, ALS–FTD had greater atrophy of the bilateral caudate.  - Compared with sporadic ALS–FTD, C9+ALS–FTD showed reduced GM volumes of the bilateral putamen, accumbens and pallidum and bilateral thalamus.  Within the entire patient cohort:  - BG and thalamic volumes were positively correlated with higher ACE- III Total scores.  - Reduced bilateral thalamic volumes were associated with poor TMT-B-A performance.  - Behavioural disturbances as odd beliefs, stereotypic and ritualistic behaviours, and apathy were negatively correlated with the volumetry of nucleus accumbens, putamen and thalamus.  - Odd beliefs were negatively associated with bilateral pallidum volumes. | - Addenbroke's Cognitive Examination (ACE- III)  -Trail Making Test (TMT)  - The Cambridge Behavioural Inventory revised (CBI-R) |
| Alruwaili et al., 2018 | - 30 ALS (17 ALScn and 13 ALSci/bi)  - 19 HC | MRI sequences:  - T1-weighted  - DTI  Technique:  - VBM  - TBSS | To compare cortical and subcortical deep GM and WM of ALS subjects and HC and to compare ALS subjects with and without cognitive impairment. | - Compared to HC, in ALS patients MD was increased in a widespread pattern of brain regions including anterior thalamic radiation.  - Compared to ALScn, ALSci/bi showed reduced GM volume in the caudate nucleus bilaterally (right>left). | - |
| Barbagallo et al., 2014 | - 24 ALS  - 22 HC | MRI sequences:  - T1-weighted  - T2-weighted  - FLAIR  Technique:  - DTI analysis | To investigate whether microstructural abnormalities occur in GM structures of the frontal-subcortical circuits in patients with ALS. | - Compared to HC, ALS patients showed higher MD in the caudate and thalamus.  - Disease duration positively correlated with the MD of the caudate and thalamus.  - Patients’ performances at the MCST and FAB negatively correlated with the MD values of the caudate. | - Modified Card Sorting Test (MCST)  - Frontal Assessment Battery (FAB) |
| Basaia et al., 2020 | - 173 sporadic ALS  - 38 PLS  - 28 PMA  - 79 HC | MRI sequences:  - T1-weighted  - T2-weighted  - FLAIR  - DTI  - RS-fMRI  Technique:  - Graph analysis, connectomics | To investigate structural and functional neural organization in ALS, PLS, and PMA. | - Compared to HC, ALS patients showed a reduced mean structural local efficiency and a longer path length within the BG network.  - Compared to HC, ALS patients presented decreased FA in BG.  In ALS patients:  - A longer path length was related to disease progression rate both at the global and lobar (within the BG network) levels.  - Functional connectivity changes (both in terms of increased and decreased functional connectivity) within the BG network and connections between BG and premotor areas correlated with disease progression.  - No correlations were observed between structural and functional brain network properties and clinical and neuropsychological variables. | - |
| Bede et al., 2013 | - 39 ALS (9 C9+)  - 44 HC | MRI sequences:  - T1-weighted  - DTI  Technique:  - Volumetry  - Vertex analysis  - ROI–based diffusion tensor data analysis | To characterize the nature and the extent of BG involvement in ALS genotypes in vivo. | *Volumetric measurements*  Compared with HC:  - C9^-^ALS patients showed volume reduction in the left caudate nucleus, right accumbens, and total BG.  - C9+ALS showed volume reductions in the bilateral thalamus, left caudate and left putamen.  *Vertex analyses*  Compared with HC:  - C9-ALS showed a reduced shape of the bilateral thalami, left caudate and bilateral accumbens nuclei.  - Compared to C9-ALS, C9+ALS showed vertex-wise differences of the right thalamus.  *ROI-based diffusivity analyses*  Compared with HC:  - C9-ALS patients showed reduced FA in the globus pallidus and increased AD, MD, and RD in the accumbens nuclei.  - Compared to C9orf72-negative patients, C9+ALS patients showed reduced thalamic FA, and increased thalamic AD, MD, and RD. | - |
| Bede et al., 2016 | - 70 ALS  - 40 HC | MRI sequences:  - T1-weighted  - DTI  Technique:  - DTI analyses  - BG Volumetry  - Vertex-wise analysis  - VBM | To investigate GM and WM changes in ALS. | - Compared to HC, the volumetric evaluation of the subcortical GM structures revealed ALS-associated atrophy of the bilateral thalamus, caudate and accumbens.  - Compared to HC, vertex analyses revealed patterns of significant atrophy in accumbens nuclei, the medial aspects of the caudate nuclei and the superior and inferior aspects of the thalami. | - |
| Bede et al., 2018 | - 10 bvFTD  - 11 nfvPPA  - 5 svPPA  - 14 C9+ALS-FTD  - 12 C9-ALS-FTD  - 36 ALScn  - 50 HC | MRI sequences:  - T1-weighted  Technique:  - Subcortical GM morphometry  - Cortical thickness | The comprehensive characterisation of subcortical GM alterations in the main FTD phenotypes along the ALS-FTD spectrum. | - Compared to HC, C9-ALS-FTD cohort showed volume reductions in the caudate nucleus, thalamus, nucleus accumbens, putamen, and pallidum.  - Compared to HC, C9+ ALS-FTD patients showed volume reductions in bilateral thalami and right accumbens.  - Compared to HC, ALScn patients showed no significant volume reductions in any of the structures. | - |
| Bharti et al., 2022 | - 120 ALS  - 115 HC | MRI sequences:  - T1-weighted  - RS-fMRI  Technique:  - Independent component analysis | To examine whole brain within-network and between-network RS-FC in a large cohort of ALS patients. | - Compared to HC, ALS patients displayed higher intra-network RS-FC in several networks including the BG network.  - In ALS patients, intra-network RS-FC in several networks including BG was negatively correlated with ALSFRS-R and with ECAS Total and sub-scores.  - Intra-network RS-FC within the BG network negatively correlated with finger and foot tapping performance.  - Compared to HC, ALS patients displayed higher inter-network RS-FC between BG and orbitofrontal networks, which was negatively correlated with the ECAS ALS specific score. | - Edinburgh Cognitive and Behavioural ALS Screen (ECAS): total, sub scores, ALS specific score |
| Branco et al., 2018 | - 50 ALS (12ALSci, 14 ALSbi)  - 38 HC | MRI sequences:  - T1-weighted  - DTI  Techniques:  - Cortical thickness  - Volumetry  - DTI analysis | To assess the brain signature of cognitive and behavioral impairment in C9orf72-negative non-de- mented ALS patients. | - ALSci group had smaller volumes of the left thalamus compared to ALScn.  - In the whole ALS cohort, a significant correlation was found between ALS-CBS-Br scores and left thalamus volumes. | - Amyotrophic Lateral Sclerosis Cognitive Behavioural Screen (ALS-CBS) |
| Buhour et al., 2017 | - 37 ALScn  - 37 HC | MRI sequences:  - T1-weighted  Technique:  - VBM | To determine both GM volume and glucose metabolism changes in a sample of patients with ALS. | - Compared to HC, ALScn patients showed GM atrophy within the right putamen. | - |
| Canna et al., 2021 | - 33 ALS  - 12 PLS  - 28 HC | MRI sequences:  - T1-weighted  - Arterial Spin Labeling  - 3D multi-echo gradient echo sequence  - FLAIR  Technique:  - Volumetry  - QSM  - ROC analyses (QSM, CBF and GM volumes as predictors) | To assess the performance of a combination of three quantitative MRI markers (iron deposition, basal neuronal metabolism, and regional atrophy) for differential diagnosis between ALS and PLS. | - Compared to HC, ALS patients showed decreased GM volumes of the left caudate.  - From the ROC analysis of combined QSM, CBF, and GM volumes, a significant discrimination of ALS patients from HC subjects was obtained in subcortical structures, including bilateral caudate, right putamen and right pallidum. | - |
| Castelnovo et al., 2021 | - 20 ALScn  - 52 HC | MRI sequences:  - T1-weighted  - FLAIR  Technique:  - Volumetry | - To identify which emotions are altered in ALS compared to HC.  - To investigate the relationship between emotion recognition and the integrity of BG, hippocampus and amygdala. | - In ALScn patients, a positive relationship was observed between the disgust correct recognition at the CATS and the GM volume of the left pallidum. | - Comprehensive Affect Testing System (CATS) |
| Castelnovo et al., 2022 | - 26 ALScn  - 52 HC | MRI sequences:  - T1-weighted  - FLAIR  - RS-fMRI  - T2-weighted  Technique:  - Seed-based RS-fMRI analysis | - To investigate the presence of altered pallidum RS-functional connectivity that can precede the pallidum structural damage in ALS  - To investigate the relationship between pallidal RS-FC changes and patients difficulties in recognising disgust. | - Compared to HC, ALScn patients showed reduced RS-FC between bilateral pallidum and bilateral middle and superior frontal and middle cingulate gyri, and increased RS-FC between bilateral pallidum and bilateral postcentral, supramarginal and superior temporal gyri and Rolandic operculum.  - Decreased RS-FC was further observed between left pallidum and left middle and inferior temporal gyri and bilateral caudate; and increased RS-FC was also shown between right pallidum and left lingual and fusiform gyri.  - In patients and HC, lower performance in recognizing disgust correlated with reduced RS-FC between left pallidum and left middle and inferior temporal gyri. | - Comprehensive Affect Testing System (CATS) |
| Chang et al., 2005 | - 10 ALScn  - 10 ALS-FTLD  - 22 HC | MRI sequences:  - T1-weighted  Technique:  - VBM | To investigate the patterns of GM and WM atrophy in ALS. | - Compared to HC, ALScn and ALS-FTLD patients showed atrophy in the left posterior thalamus. | - |
| Chipika et al., 2020 | - 100 ALS (12 C9+ALS, 88 C9-ALS)  - 33 PLS  - 117 HC | MRI sequences:  - T1-weighted  Technique:  - Vertex analyses  - Region-of-interest morphometry | To characterize the integrity of thalamic nuclei in patients with ALS and PLS. | - Compared to HC, all ALS patients exhibited volume reduction in the mediodorsal-paratenial-reuniens group of nuclei.  - Compared to HC, C9-ALS patients exhibited significant volume reductions in motor, sensory and intralaminar, anteroventral, medial geniculate, and lateroposterior thalamic nuclei.  - No associations between the volumes of thalamic nuclei and clinical variables (ALSFRS-R and symptom duration) were observed. | - |
| Christidi et al., 2018a | - 56 ALS  - 25 HC | MRI sequences:  - T1-weighted  - DTI  - FLAIR  Technique:  - VBM  - TBSS | To examine the neuroanatomical substrate of pathological laughing and crying in a sample of patients with ALS. | - Compared to HC, ALS patients showed decreased GM volumes of thalamus and putamen.  - Compared to ALS without pathological laughing and crying, patients with pathological laughing and crying showed decreased left putamen volume and decreased FA of the left anterior thalamic radiation. | - |
| Christidi et al., 2018b | 19 ALScn  31 ALS-ci  25 HC | MRI sequences:  - T1-weighted  - DTI  - FLAIR  Technique:  - VBM  - TBSS | To investigate both GM and WM changes in non-demented ALS patients with or without cognitive impairment. | - Compared to HC, ALSci patients showed diffuse GM volume reduction in several clusters located in the left caudate and putamen. | - |
| Cividini et al., 2021 | - 83 sporadic ALS (54 ALScn, 21 ALS-ci/bi, 8 ALS-FTD)  - 35 sporadic bvFTD  - 61 HC | MRI sequences:  - T1-weighted  - T2-weighted  - FLAIR  - DTI  - RS-fMRI  Technique:  - Graph analysis, connectomics | To investigate structural and functional network correlates of cognitive/behavioral impairment in patients within the ALS-FTD continuum. | - Compared to HC, ALScn patients presented a more focal structural damage within the sensorimotor-BG areas;  - Compared to HC, ALSci/bi patients demonstrated the same structural damage of ALScn patients, together with enhanced FC within sensorimotor areas and decreased FC within frontotemporal and parietal networks;  - Compared to HC, ALS-FTD patients showed both structural and functional disruption of the frontotemporal and parietal networks, and the typical ALScn structural damage within the sensorimotor-BG areas. | - |
| De Vocht et al., 2020 | - 17 presymptomatic carriers of C9orf72 repeat expansion  - 29 HC | MRI sequences:  - T1-weighted  - T2-weighted  Technique:  - Voxel–based volumetric analysis | To evaluate metabolic and structural changes before symptom onset in presympromatic carriers of C9orf72 expansion. | - Compared to HC, presymptomatic carriers of C9orf72 repeat expansion showed significant clusters of reduced GM volume of BG and thalami. | - |
| Fekete, et al., 2013 | - 40 ALS  - 30 HC | MRI sequences:  - RS-fMRI  Technique:  - Motor network correlation analysis  - Support vector machine approach with recursive kernel elimination  - Complex network analysis | To test the hypothesis that a systems-level signature capturing the core of ALS pathology, despite its inherent clinical and prognostic heterogeneity, might be identifiable using RS-fMRI data. | - Compared to HC, ALS patients exhibited clusters of increased connectivity in the BG. | - |
| Finegan et al., 2019 | - 33 PLS  - 100 ALS  - 117 HC | MRI sequences:  - T1-weighted  Technique:  - Volumetry | To identify subcortical signatures that may distinguish PLS from ALS. | - Compared to HC, ALS patients showed significant volume reductions in the bilateral thalamus, left caudate and right accumbens. | - |
| Kim et al., 2017 | - 47 sporadic ALS  - 28 HC | MRI sequences:  - T1-weighted  Technique:  - VBM | To investigate the specific patterns of brain atrophy in sporadic ALSci patients using VBM. | - Compared to ALScn, ALSci patients showed reduced GM volume of the right putamen. | - |
| Konrad et al., 2006 | - 10 ALS  - 10 HC | MRI sequences:  - T1-weighted  - Task-based fMRI (simple finger flexion task)  Technique:  - Whole brain fMRI analysis | To test the hypothesis that, in addition to the known cortical changes, degenerative cortical and spinal motor neuron lesions in ALS also lead to subcortical reorganization. | - Compared to HC, ALS patients showed increased activation within the BG, especially in the putamen. | - |
| Langkammer et al., 2010 | - 15 ALS  - 15 HC | MRI sequences:  - T1-weighted  - FLASH  - DTI  Technique:  - TBSS | To test if and where increased iron accumulation occurs in ALS and to relate these findings to WM tract degeneration assessed by DTI. | - Compared to HC, ALS patients showed increased iron deposition in the caudate nucleus, reduced FA and increased MD in the globus pallidus and the putamen.  - No relation was found between iron deposition and WM in BG regions. | - |
| Leoni et al., 2022 | - 10 presymptomatic carriers of the Pro56Ser VAPB variant  - 20 symptomatic carriers of the Pro56Ser VAPB variant  - 30 HC  - 20 sporadic ALS | MRI sequences:  - T1-weighted  - T2-weighted  - DTI images  Technique:  - Cortical thickness  - BG volumetry  - WM analysis  - Spinal cord morphometry | To evaluate structural brain and spinal cord abnormalities in symptomatic and pre-symptomatic VAPB-related ALS. | - Compared to HC, symptomatic carriers of the Pro56Ser VAPB variant showed significant atrophy in the globi pallida. | - |
| Li et al., 2022 | - 34 ALS  - 34 HC | MRI sequences:  - T1-weighted  Technique:  - VBM  - QSM | To investigate iron deposition, GM atrophy, and their associations with disease severity in motor cortex and thalamus in ALS. | - ALS patients showed increased iron deposition and reduced GM volume of bilateral thalamus.  - In ALS, a negative correlation between thalamic iron deposition and ALSFRS-R was observed. | - |
| Liu et al., 2021 | - 76 ALS  - 94 HC | MRI sequences:  - T1-weighted  - DTI  - FLAIR  Technique:  - Volumetry of hippocampus and thalamus  - Probabilistic tractography approach | - To investigate in vivo, the degree to which hippocampal subfield volumes differ between ALS patients at different stages, using the thalamus, corticostriatal tract and perforant pathway as structural controls. | - Compared with HC and ALS patients at King’s stages 1 and 2, ALS patients at King’s stage 3 had greater atrophy of the bilateral thalamus. | - |
| Ma et al., 2016 | - 20 ALS  - 20 HC | MRI sequences:  - RS-fMRI  - T1-weighted  Technique:  - RS-fMRI  - ALFF | To determine whether the abnormalities of fALFF in ALS are associated with specific frequency bands and whether the alterations of fALFF on specific bands are correlated with ALS clinical manifestations. | - Compared to HC, ALS patients showed increased fALFF in the right caudate nucleus.  - No significant correlation between fALFF and clinical measures (ALSFRS-R, disease duration, and disease progression rate) was determined. | - |
| Machts et al., 2015 | - 67 ALS (7 ALS-FTD, 18 ALSci/bi and 42 ALScn)  - 39 HC | MRI sequences:  - T1-weighted  - T2-weighted  - FLAIR  Technique:  - Volumetry, shape and density analyses | To evaluate BG changes along the ALS-FTD continuum using multiple, complementary imaging techniques. | - Compared with all other groups the ALS-FTD group presented widespread GM atrophy affecting several regions including BG and thalamus. The anatomical pattern was similar among groups, however the extent of atrophy observed in ALS-FTD was more marked in comparison to ALScn than in contrast to ALSci/bi.  - In all ALS patients, accumbens nuclei volumes showed a negative correlation with apathy scores. | - |
| Masuda et al., 2016 | - 51 ALS (19 ALScn and 25 ALSci, 7 ALS-FTD)  - 24 HC | MRI sequences:  - T1-weighted  - DTI  - FLAIR  Technique:  - VBM  - TBSS | - To assess specific and common features of cortical and subcortical GM and WM involvement in ALS patients with and without cognitive decline.  - To identify structural networks associated with cognitive decline in ALS. | - Compared to HC, ALS-FTD patients showed reduced GM volume in the caudate head, thalamus and putamen  - Compared to HC, ALSci patients showed reduced GM volume in the caudate head.  - Compared to HC, patients with ALSci and ALS-FTD showed widespread decreased FA of the superior thalamic radiation, areas surrounding the head of caudate nucleus and the stria terminalis.  - The probabilistic diffusion tractography of the head of the caudate nucleus demonstrated that the structural connectivity between the caudate nucleus head and the dorsomedial frontal lobe were markedly retracted in all patients, even in patients with ALScn, compared with HC. | - |
| Menke et al., 2014 | - 60 sporadic ALS  - 36 HC  Longitudinal assessment: 6 months | MRI sequences:  - T1-weighted  - DTI  Technique:  - VBM  - TBSS | To assess GM and WM longitudinal changes in ALS. | - The longitudinal VBM analysis showed GM volume decreases encompassing widespread areas including thalami and caudate heads bilaterally in ALS patients compared to HC. | - |
| Menke et al., 2014 | - 13 ALS  - 3 PLS  Longitudinal assessment: 24 months | MRI sequences:  - T1-weighted  - DTI  - RS-fMRI  Technique:  - VBM  - Volumetry  - TBSS  - RS-fMRI analysis (indepedent component analysis) | To identify the extent of structural and functional MRI changes detectable over a longer period of follow-up. | Over 24 months, ALS patients showed:  - Atrophy of the thalamus, caudate, pallidum bilaterally and of the right putamen.  - FA decreases in the right thalamus.  - Increased RD, AD and MD in the GM of the left and right caudates and thalami.  - FC decreases between a network comprising both thalami and an area in the visual cortex in relation to both time and ALSFRS-R decline. | - |
| Mohammadi et al., 2015 | - 17 ALS  - 17 HC | MRI sequences:  - T1-weighted  - RS-fMRI  - Task-based fMRI (a visual stop-signal task).  Technique:  - Whole brain fMRI analysis | To expand on the research on impaired inhibitory control in ALS. | - ALS patients showed increased inhibition-related activity in putamen and pallidum. | - |
| Mohammadi et al., 2009 | - 22 ALS (10 with bulbar signs, 12 without bulbar signs)  - 5 Kennedy syndrome | MRI sequences:  - T1-weighted  - RS-fMRI  - Task-based fMRI (vertical movements with the tongue)  Technique:  - fMRI analysis | To investigate cortical activity during movements of the tongue to delineate the neurofunctional correlates of bulbar symptoms. | - Compared to HC, ALS patients with bulbar signs activated less the thalamus during vertical tongue movements. | - |
| Sach et al., 2004 | -15 ALS (including six patients developing clinical signs of UMN lesion after the time of MRI investigation)  -12 HC | MRI sequences:  - T1-weighted  - DTI (STEAM sequence)  Technique:  - VBM on WM | To study WM changes in the motor system in ALS. | - In ALS patients and in six patients without any clinical signs of UMN lesion at the time of MRI investigation, FA was reduced in the right thalamus. | - |
| Senda et al., 2017 | - 67 ALS (19 with slow progression, 36 with intermediate progression, 12 with rapid progression)  - 38 HC  Longitudinal assessment: 6 months | MRI sequences:  - T1-weighted  - DTI  Technique:  - VBM  - TBSS | To examine whether the extent of MRI brain alteration observed at first visit is related to the different disease progression of ALS. | - In the rapid progression ALS group, GM volume reductions were more widespread and severe than in slow and intermediate progression groups. The regions involved the caudate head and thalamus.  - All three ALS groups showed lower FA beyond extramotor regions, including the region surrounding the caudate nucleus and thalamus. Compared to the slow progression group, the rapid progression group showed a more severe and widespread pattern of damage. | - |
| Sharma et al., 2012 | - 14 ALS  - 12 HC | MRI sequences:  - T1-weighted  - T2-weighted  - FLAIR  - DTI  Technique:  - DTI analysis | To assess the involvement of BG and thalamus in patients with ALS using DTI. | - Compared to HC, in ALS patients showed increased MD, AD and RD, and reduced FA in caudate, putamen, globus pallidus and thalamus.  In ALS patients:  - Maximum rate of finger-tap was positively correlated with FA values of globus pallidus.  - Maximum rate of foot-tap was positively related to FA values of caudate, globus pallidus, and negatively related to MD, AD, and RD of globus pallidus.  - The maximum bulbar muscle-movement rates of syllable repeats was positively related to FA values of putamen and negatively related to AD values of globus pallidus.  - The ALSFRS-R score negatively correlated with the MD values of putamen.  - The MoCA score negatively correlated with the MD values of caudate. | - Montreal Cognitive Assessment  (MoCA) |
| Spinelli et al., 2021 | - 44 MND (45 sporadic, 44 genetic)  - 22 FTD (16 sporadic, 22 genetic) | MRI sequences:  - T1-weighted  - T2-weighted  Technique:  - VBM  - Volumetry | To explore the neuroanatomical structural correlates of genetic heterogeneity in a cohort of patients affected by the wide spectrum of FTLD disorders, including MND. | *VBM analysis:*  - Compared with HC, patients with C9+MND showed atrophy of several regions including left thalamus.  - Compared with patients with sporadic MND, patients with C9+MND showed greater atrophy of several regions including right posterior thalamus.  *Volumetric GM analysis:*  - Compared with HC, patients with C9+MND showed significant volumetric reduction of the caudate nuclei and thalami.  - Compared with patients with sporadic MND, C9+MND patients showed significant volumetric reduction of the thalami, bilaterally and left caudate. | - |
| Tae et al., 2020 | - 32 ALScn  - 43 HC | MRI sequences:  - T1-weighted  Technique:  - Surface-based vertex analysis of the subcortical nuclei | To investigate local shape changes and mean volumes of the subcortical nuclei in sporadic ALS patients with preserved cognition. | - Compared to HC, ALS patients showed regional shape contractions that suggested local atrophy in both pallida, right putamen, and right nucleus accumbens.  - Disease progression rate was negatively correlated with the local shape distances in the bilateral putamen. | - |
| Gorno-Tempini et al., 2021 | - 69 HC  - 97 ALS (68 ALScn; 21 ALSci; and 8 ALS-FTD) | MRI sequences:  - RS-fMRI  - T1-weighted  Technique:  - ROI based fMRI analysis (seeds: motor cortex for motor network, posterior cingulate cortex for DMN, ventral striatum for ventral attention network) | To investigate RS functional connectivity differences between ALScn, ALSci, and ALS-FTD. | - Compared to HC, ALS patients showed a widespread pattern of decreased functional connectivity in all three networks.  - Worse TMT B-A performance was associated with increased connectivity in the left thalamus and putamen within the ventral attention network. | - Trail Making Test (TMT) |
| Tessitore et al., 2006 | - 16 sporadic ALS  - 13 HC | MRI sequences:  - Task-based fMRI (simple visually paced motor task)  Technique:  - Whole brain fMRI analysis | To address the potential contribution of BG activity in the motor system functional reorganization during the performance of ALS patients at a simple visually paced motor fMRI task. | - Compared to HC, during the task ALS patients (in particular those with greater UMN involvement) showed increased activation of the left anterior putamen and right caudate. | - |
| Thivard et al., 2017 | - 15 ALS  - 25 HC | MRI sequences:  -T1-weighted  -FLAIR  -Diffusion weighted spin echo  Technique:  -Voxel based diffusion data analysis  -VBM | To investigate the extent of cortical and  subcortical lesions in ALS using, in combination, DTI and VBM. | - Compared to HC, ALS patients showed reduced GM volume and FA values of thalamus bilaterally. | - |
| Trojsi et al., 2019 | - 36 ALS (ALScn; 4 ALSci; 6 ALSbi; 1 ALSci/bi)  - 35 HC | MRI sequences:  - DTI  - FLAIR  Technique:  - TBSS  - Volume of interest analysis | To investigate WM microstructural alterations associated with ECAS performances in a cohort of non-demented ALS patients. | - The total ALS non-specific score was inversely related to AD values in the left medio-dorsal nucleus of the thalamus.  - Moreover, negative correlations were reported between the isolated memory subscore and MD/RD measures in the left mediodorsal nucleus of the thalamus. | - Edinburgh Cognitive and Behavioural ALS Screen (ECAS): ALS non-specific score |
| Tu et al., 2018 | - 20 ALS  - 31 HC | MRI sequences:  - T1-weighted  - DTI  Technique:  - Vertex analysis tractography | To assess the integrity of the thalamus and its connectivity to the major cortical regions of the brain in a longitudinal manner. | - Significant bilateral reduction in total thalamic volume in ALS compared with HC.  - Increased diffusion in thalamic parcellations connected with the left frontal lobe, bilateral premotor cortex, bilateral motor cortex, right somatosensory cortex and bilateral parietal lobe, in patients with ALS compared with HC. | - |
| Walhout et al., 2015 | - 16 asymptomatic C9orf72 carriers  - 23 noncarriers from C9orf72 family  - 14 C9+ALS  - 28 HC | MRI sequences:  - T1-weighted  - DWI  Technique:  - Cortical thickness  - Volumetry of subcortical regions, ventricles and cerebellum | To investigate possible effects of the C9orf72 repeat expansion before disease onset on brain morphology. | - Analysis of subcortical regions showed volume reduction of the left caudate and left putamen in asymptomatic carriers compared with noncarriers.  - Compared to HC, C9+ALS patients presented with smaller volumes of bilateral thalamus and putamen, right caudate, left pallidum and nuclei accumbens. | - |
| Westeneng et al., 2015 | - 112 ALS (7 C9+ALS)  - 60 HC  Longitudinal assessment: median time 5.5 months | MRI sequences:  - T1-weighted  Technique:  - Volumetry  - Vertex-based shape analyses | To assess the involvement of deep GM, hippocampal subfields, and ventricular changes in patients with ALS. | - The shape analysis revealed that ALS patients compared to HC presented regional volume change in both thalami.  - Smaller BG were associated with a shorter survival. | **-** |
| Westeneng et al., 2016 | - 156 sporadic ALS  - 14 C9+ALS | MRI sequences:  - T1-weighted  - DWI  Technique:  - Region-wise and whole-brain vertex-wise cortical thickness  - Logistic regression | To investigate the specificity of C9orf72 repeat expansion in patients with ALS. | - Compared with C9-patients, C9+ALS patients showed smaller volumes of the bilateral thalamus, caudate nucleus, putamen, and nucleus accumbens.  - From the logistic regression emerged that the ‘C9+’ neuroimaging phenotype was defined by eleven variables including the volume of the thalamus and nucleus accumbens. These 11 imaging variables enabled accurate classification of patients (95.2%) into C9+ or C9−. | - |
| Xu et al., 2017 | - 20 ALS  - 21 HC | MRI sequences:  - RS-fMRI  Technique:  - Graph analysis | To investigate the functional abnormalities of the cortico-BG network in ALS. | - Compared to HC, ALS patients exhibited decreased nodal efficiency in the right thalamus, left caudate and right precentral gyrus.  - No correlations were observed between the nodal efficiency of abnormal brain regions and the clinical measurements (disease duration, ALSFRS-R, and disease progression rate). | - |
| Zhang et al., 2016a | - 38 ALS  - 35 HC | MRI sequences:  - DTI  - RS-fMRI  Technique:  - Voxel mirrored homotopic connectivity  - Probabilistic fiber tracking | - To examine alterations of interhemispheric structural and functional connectivity in ALS. | - Compared to HC, ALS patients showed reduced voxel mirrored homotopic connectivity in the putamen. | - |
| Zhang et al., 2017b | - 38 ALScn  - 35 HC | MRI sequences:  - DTI  Technique:  - Probabilistic fiber tracking | - To study the selective vulnerability of different thalamocortical structural connections in ALS.  - To test the hypothesis of a specific impairment in motor-related thalamocortical connectivity. | - FA values of the connection between bilateral premotor and motor pathways and bilateral thalamus were significantly decreased in ALS patients compared to HC.  - Inverse correlation between FA of the tract connecting the thalamus and bilateral motor cortices and the disease duration.  - Inverse correlation between FA of the tract connecting the thalamus and bilateral M1 cortices and the disease duration. | - |

**Abbreviations:** ALFF=amplitude of low-frequency fluctuation; ALS=amyotrophic lateral sclerosis; ALS-CBS-Br=Brazilian Portuguese version of the Amyotrophic Lateral Sclerosis Cognitive Behavioural Screen; ALSci/bi=ALS with cognitive/behavioural deficits; ALScn=ALS cognitively normal; ALS-FTD=ALS with frontotemporal dementia; AD=axial diffusivity; BG=basal ganglia; BOLD= Blood-oxygen-level-dependent imaging; bvFTD=behavioural variant of frontotemporal dementia; DMN=Default Mode Network; C9+ALS=ALS patients carrying C9orf72 mutation; C9-ALS=ALS patients not carrying C9orf72 mutation; DTI=diffusion tensor imaging; FA=fractional anisotropy; FC=functional connectivity; FDG= Fluorodeoxyglucose; FIRST=FMRIB’s Integrated Registration and Segmentation Tool; FLAIR= Fluid-attenuated inversion recovery; fMRI=functional MRI; FSL=FMRIB Software Library; FTD= Frontotemporal dementia; FTLD=Frontotemporal lobar degeneration; GM=grey matter; HC=healthy controls; MD=mean diffusivity; MRI=magnetic resonance imaging; PET=Positron Emission Tomography; RD=radial diffusivity; ROI=region of interest; RS-fMRI=resting-state fMRI; SMA= supplementary motor area; TBSS=Tract-Based Spatial Statistics; QSM= Quantitative susceptibility mapping; VBM=Voxel-based Morphometry; WM=white matter; [18F]FDG-PET= fluorine 18–labeled fluorodeoxyglucose positron emission tomographic.
